# Morphological image processing for quantitative shape analysis of biomedical structures: effective contrast enhancement

**DOI:** 10.1107/S0909049513020761

**Published:** 2013-09-25

**Authors:** Yoshitaka Kimori

**Affiliations:** a Imaging Science Division, Center for Novel Science Initiatives, National Institutes of Natural Sciences, 5-1 Higashiyama, Myodaiji, Okazaki, Aichi 444-8787, Japan

**Keywords:** image processing, contrast enhancement, mathematical morphology

## Abstract

Image processing methods significantly contribute to visualization of images captured by biomedical modalities (such as mammography, X-ray computed tomography, magnetic resonance imaging, and light and electron microscopy). Quantitative interpretation of the deluge of complicated biomedical images, however, poses many research challenges, one of which is to enhance structural features that are scarcely perceptible to the human eye. This study introduces a contrast enhancement approach based on a new type of mathematical morphology called rotational morphological processing. The proposed method is applied to medical images for the enhancement of structural features. The effectiveness of the method is evaluated quantitatively by the contrast improvement ratio (CIR). The CIR of the proposed method is 12.1, *versus* 4.7 and 0.1 for two conventional contrast enhancement methods, clearly indicating the high contrasting capability of the method.

## Abstract 2

A contrast enhancement approach utilizing a new type of mathematical morphology called rotational morphological processing is introduced. The method is quantitatively evaluated and then applied to some medical images.

## 1. Introduction

Image processing is a crucial step in the quantification of biomedical structures from images. As such, it is fundamental to a wide range of biomedical imaging fields. Image processing derives structural features, which are then numerically quantified by image analysis. Contrast enhancement plays an important role in image processing; it enhances structural features that are hardly detectable to the human eye and allows automatic extraction of those features. Numerous contrast enhancement methods have been reported in the literature (Wang & Vagnucci, 1981 ▶; Pisano *et al.*, 2000 ▶; Galatsanos *et al.*, 2003 ▶), including histogram modification methods, and spatial domain and frequency domain filtering (Gonzalez & Woods, 2008 ▶). However, these methods tend to enhance entire structures in a biomedical image without discrimination. To effectively recognize a region of interest, specific target structures must be enhanced while surrounding objects remain unmodified.

One contrast enhancement technique uses mathematical morphology, which enables selective enhancement of target structures. Based on set theory, mathematical morphology applies shape information to image processing (Serra, 1982 ▶). Several morphological contrast enhancement methods have been proposed and applied to medical images (Thackray & Nelson, 1993 ▶; Kobatake & Yoshinaga, 1996 ▶; Reljin *et al.*, 2009 ▶; Oh & Hwang, 2010 ▶).

Mathematical morphology operates by a series of morphological operations, which use small images called structuring elements (typically, a single structuring element is used). The structuring element acts as a moving probe that samples each pixel of the image. Since the structuring element moves in a fixed direction across the image, some intricate images (in particular, those whose structural details contain a variety of directional characters) may not be properly processed. Consequently, an artifact in the shape of structuring elements may be generated at the object periphery. Since objects in biomedical images consist of delicate structural features, this drawback is an especially serious problem.

To overcome this problem, we have proposed an extension of conventional mathematical morphology called rotational morphological processing (RMP) (Kimori *et al.*, 2007 ▶, 2010 ▶). Morphological filters based on this method have been applied to a wide variety of biomedical images, including electron micrographs (Kimori *et al.*, 2007 ▶, 2011 ▶), light micrographs (Kimori *et al.*, 2010 ▶; Chisada *et al.*, 2011 ▶; Yasuda *et al.*, 2012 ▶) and medical images such as mammographic images and chest X-ray images (Kimori, 2011 ▶).

This study describes an RMP-based contrast enhancement method. The method uses a top-hat contrast operator (Meyer, 1979 ▶), a well known and commonly used morphological operation for extracting local features from a low-contrast image. Two types of top-hat operations exist: white top-hat (WTH) and black top-hat (BTH). WTH and BTH extract structures brighter and darker than the surrounding areas, respectively. In the proposed method, these RMP-based top-hat operators are computed in parallel. The method operates in three main steps: (i) selective extraction of target features by the top-hat operations, (ii) greyscale modification of the top-hat images, and (iii) combining greyscale modified top-hat images with the original image. This method specifically enhances target structures from complex surrounding structures and uneven background intensity.

In this study, the method is subjectively and quantitatively evaluated by the contrast improvement ratio (CIR) (Wang *et al.*, 2003 ▶), using a synthetic image. It is then applied to two real medical images: a mammographic image and a chest radiographic image. The efficiency of the method is clearly demonstrated.

This paper is organized as follows: the current section presents relevant background information. The sample images and methodology are provided in the *Methods* section[Sec sec2], while results are presented in the *Results and discussion* section[Sec sec3]. The final section presents the study conclusions[Sec sec4].

## 2. Methods

### 2.1. Sample images

The performance of the proposed method was assessed on two types of medical images: mammographic and chest radiographic images. Mammographic images were obtained from the mini mammography database provided by the Mammographic Image Analysis Society (Suckling *et al.*, 1994 ▶). The size of each mammographic image was 1024 × 1024 pixels with a spatial resolution of 200 µm pixel^−1^.

Chest radiographic images were obtained from the standard digital image database for chest lung nodules and non-nodules provided by the Japanese Society of Radiological Technology (Shiraishi *et al.*, 2000 ▶). The image size was 2048 × 2048 pixels with a spatial resolution of 0.175 mm pixel^−1^.

### 2.2. Overview of proposed method

**Figure 1:**
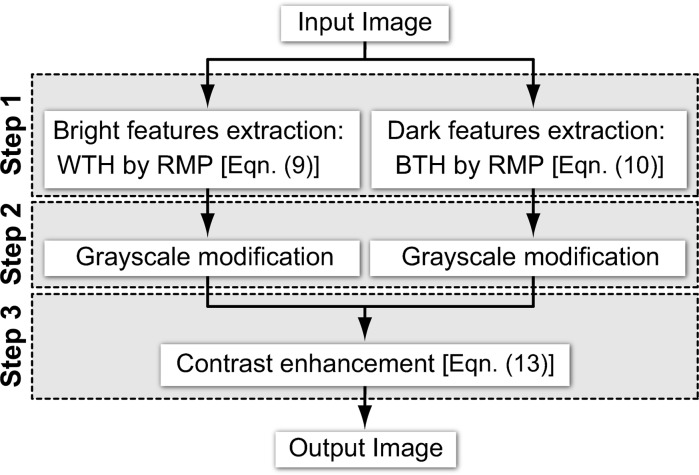
Flow diagram of the proposed contrast enhancement method (WTH and BTH denote white top-hat and black top-hat, respectively; RMP = rotational morphological processing).

Fig. 1 ▶ shows a flow diagram of the proposed method. The overall process consists of three steps: (1) selective extraction of target features by top-hat operations, in which bright and dark features are extracted by white and black top-hats, respectively; (2) greyscale modification of the top-hat images, using two consecutive greyscale modification techniques; (3) combination of the original and greyscale modified top-hat images.

### 2.3. Conventional morphological image processing

Mathematical morphology (Serra, 1982 ▶) is a powerful tool for extracting structural characteristics in an image and is useful for characterizing shape information. Mathematical morphology is based on set theory. An image is regarded as a set (binary image) or a function (greyscale image), acted upon by a set of nonlinear operators using structuring elements. The structuring element, which indicates the shape characteristics in an image, is generally a small and simple binary image, such as a disc, square or line segment. The two basic morphological operators are dilation and erosion. Dilation (defined as a maximum operator) selects the brightest value in a neighbourhood of the structuring element. Erosion (minimum operator) is the dual operation of dilation, *i.e.* it selects the darkest value in a neighbourhood. Many other operations are derived from these operations. Given a greyscale image *f* and a structuring element *B*, dilation and erosion are defined as follows:
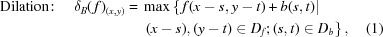


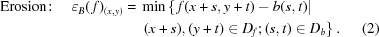
Here, (*x*, *y*) and (*s*, *t*) are the coordinate sets of the images *f* and *B*, respectively. Also, *D*_*f*_ and *D*_*b*_ are the domains of the functions *f* and *B*, respectively. These operations are applied in tandem using the following opening and closing operations,
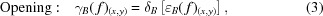


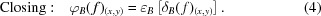
Opening and closing operate on features smaller than the width of the structuring element. Opening is defined as erosion followed by dilation within the same structuring element. Closing is dual to opening, *i.e.* dilation followed by erosion within the same structuring element. The opening operation removes bright features (*e.g.* peaks and ridges), while the closing operation fills in dark regions (*e.g.* troughs, holes and gaps). By virtue of their characteristics, these operations lead to smooth processing and are combined to produce morphological filters of various capabilities.

Top-hat transform is a well known and commonly used morphological operation for locally extracting structures from given images (Meyer, 1979 ▶). Structural extraction is unrelated to grey level correction and is independent of background brightness. As such, it allows for the extraction of target structures from an uneven background. Recall that the WTH and BTH operators extract bright and dark features, respectively. In both cases the sizes of extracted structures are smaller than that of the structuring element. The WTH subtracts the opening image γ_*B*_(*f*) with structuring element *B* from the original greyscale image *f*,
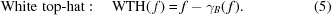
WTH yields an image containing all the residual structures (*e.g.* peaks and ridges) subtracted by the opening operation. The sizes and shapes of the extracted structures are determined by the size and shape of the structuring element.

By contrast, BTH subtracts the original greyscale image *f* from the closing image ϕ_*B*_(*f*) encased within structuring element *B*,
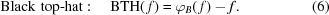
BTH extracts the dark regions (*e.g.* troughs, holes and gaps) filled by the closing operation. Similarly to WTH, the sizes and shapes of the extracted regions are determined by the size and shape of the structuring element.

If the greyscale levels of the target structures and background in an image are similar, the target structures are not easily detected by the human eye. However, these low-contrast targets can be extracted and enhanced by the top-hat transforms.

### 2.4. Rotational morphological processing

As previously mentioned, conventional morphological operations apply the structuring element in a fixed direction during a given process. Such unidirectional fitting of the structuring element to image structures limits the applicability of the technique to images containing simple directional structures. Images containing more complex directional variations (including biomedical images) are prone to artifacts caused by insufficient morphological processing.

In RMP (Kimori *et al.*, 2007 ▶, 2010 ▶), the morphological filters enable isotropic processing using a single structuring element. The RMP algorithm subjects a single structuring element to a series of rotations of the original image. Conventional morphological operators (*i.e.* opening and closing) are applied to each rotated image. Finally, the processed images are combined into an output image using a maximum or minimum operator of image pixel value.

The method is detailed by Kimori *et al.* (2007 ▶, 2010 ▶). The steps of the procedure are as follows:

Step 1. The original image *f* is rotated clockwise (with respect to the centre of the image frame). A half circle (π [rad]) is equally divided into *N* directions. Let *f*_*i*_ denote the rotation of an original image *f* by angle θ_*i*_ = π*i*/*N* [rad], where *i* = 0, 1,…, *N* − 1.

Step 2. The rotated images are processed (by opening or closing operations) using a single structuring element *B*. The results of opening and closing operations on the rotated image *f*_*i*_ within *B* are denoted γ_*B*_(*f*_*i*_) and ϕ_*B*_(*f*_*i*_), respectively.

Step 3. The processed images are rotated counterclockwise by θ_*i*_ [rad]. The *i*th rotated opened and closed images are denoted 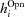 and 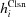, respectively.

Step 4. The processed images are combined into the final output. RMP opening (closing) processing creates the output image each of whose pixels stores the maximum (minimum) value over all processed images at corresponding pixel location.

Under RMP, the opening 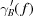 and closing 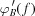 operators are defined as follows,
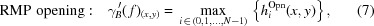


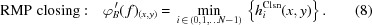
When *N* = 1, the above operations are equal to the conventional operations. The choice of *N* depends upon the shape of the structuring element. The optimal *N* for a disc or square structuring element is 8, whereas that of a line-segment structuring element is 36 (Kimori *et al.*, 2010 ▶). RMP significantly reduces the artifacts induced by the unidirectional raster of a single structuring element. For example, when RMP is applied to morphological smoothing of a noisy image, noise is satisfactorily reduced while details and edges are preserved (Kimori *et al.*, 2007 ▶, 2010 ▶).

In terms of RMP, the top-hat operators are formulated as follows,
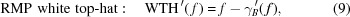


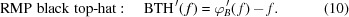
These RMP-based top-hat operations have already been applied to various biomedical structures. We have extracted bright spots from fluorescence microscope images using modified WTH (Kimori *et al.*, 2010 ▶). Modified WTH is also applicable to clustered or aggregated spots. RMP-based BTH has been used to extract the surface structural pattern on an actin filament, which is the main component of the cyto­skeleton captured by electron microscopy (Kimori *et al.*, 2011 ▶).

### 2.5. Top-hat contrast enhancement operator based on RMP

The top-hat contrast enhancement operator κ is implemented by parallel computing of WTH and BTH. WTH is added to the original image to enhance bright structures while BTH is subtracted from the resulting image to enhance dark structures (Soille, 1999 ▶). This operator is defined as follows,
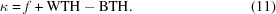
Similarly, we define the RMP-based top-hat contrast operator as
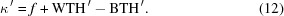
Low-contrast target structures cannot be enhanced by top-hat operations because the greyscale values of such operations are too small to contribute meaningfully to contrast enhancement. Therefore, enhancement of low-contrast structures cannot be achieved by operations (11) and (12) alone.

To overcome this difficulty, an adequate greyscale modification technique is required. Two greyscale modification techniques are introduced to the top-hat contrast operation. More specifically, following selective extraction of the structures to be enhanced by top-hat operations, contrast of the extracted features is enhanced by two greyscale modification techniques. Contrast in the top-hat image is enhanced by histogram equalization (Gonzalez & Woods, 2008 ▶) followed by linear contrast stretching (Gonzalez & Woods, 2008 ▶). The former technique manipulates the histogram of an image by redistributing the number of pixels of different intensities to equal frequency. The latter normalizes the greyscale levels by determining the minimum and maximum greyscale values in an image and then linearly stretching this range so that the full range is available for output intensities (*e.g.* 0–255 for an 8-bit greyscale image).

WTH′- and BTH′-based contrast enhancement (*i.e.* WTH′ and BTH′ followed by both greyscale modification techniques) are denoted ν_WTH′_ and ν_BTH′_, respectively.

The proposed contrast enhancement operator λ is formulated in terms of ν_WTH′_ and ν_BTH′_ as follows,
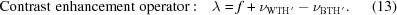
Once ν_WTH′_ and ν_BTH′_ have been combined with the original image, the greyscale values usually fall below or above the dynamic range. Thus, the output greyscale range of λ is set to the image display range (*e.g.* [0:255] for an 8-bit greyscale image).

In the proposed method, the images processed by WTH′ and BTH′ are enhanced in turn, with a high degree of contrast enhancement expected.

### 2.6. Contrast improvement ratio

Contrast improvement ratio (CIR) measures the effect of contrast enhancement on image quality (Wang *et al.*, 2003 ▶). CIR is defined as the ratio of the contrast of the enhanced and unenhanced images within region of interest *R*, as follows,
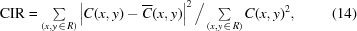
where *C*(*x*, *y*) and 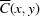 are the local contrast values at (*x*, *y*) of the unenhanced and enhanced images, respectively. The local contrast value *C*(*x*, *y*) is computed as
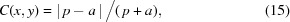
where *p* and *a* are the mean contrasts within the centre region (*R*_C_) and the neighbourhood region (*R*_N_), respectively.

## 3. Results and discussion

### 3.1. Evaluation of contrast enhancement on a synthetic test image

**Figure 2:**
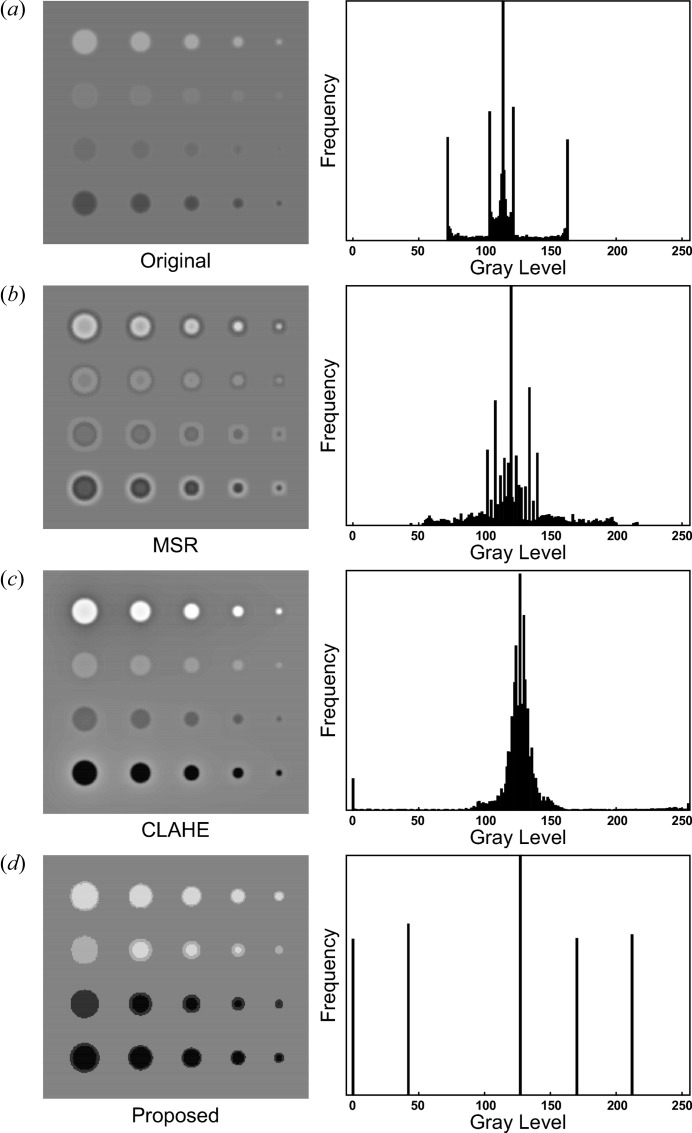
Comparison of contrast enhancement methods on a synthetic test image. (*a*) Synthetic test image (left) and its histogram of grey level distributions (right). (*b*)–(*d*) Contrast enhancement images and their corresponding histograms obtained by the following methods: multiscale retinex (*b*), contrast-limited adaptive histogram equalization (*c*), the proposed method (*d*).

To evaluate contrast improvement by the proposed method, the method was compared with two conventional contrast enhancement methods: multiscale retinex (MSR) (Jobson *et al.*, 1997 ▶) and contrast-limited adaptive histogram equalization (CLAHE) (Pizer *et al.*, 1987 ▶). Evaluation was conducted on a synthetic test image of size 238 × 218 pixels. The image enhancements achieved by the proposed and conventional methods are presented in Fig. 2 ▶. Fig. 2(*a*) ▶ shows the synthetic test image and the corresponding histogram of greyscale intensity distributions. Fig. 2(*b*) ▶ shows the enhancement result obtained by MSR, which adopts typical retinex theory for image contrast enhancement and calculates the output image values as the difference between the original image and its blurred version in the logarithmic domain. For this technique, three convolution scales were used with standard deviations set to 5, 15 and 25 pixels. The weights were set to 1/3 for each scale.

Fig. 2(*c*) ▶ shows the enhancement output by CLAHE, an adaptive image contrast enhancement technique based on histogram modification. This method operates on small regions (blocks) in an image. In this investigation the block size was set to 9 × 9 pixels.

Fig. 2(*d*) ▶ shows the enhancement generated by the proposed contrast enhancement operator λ, assuming a disc-shaped structuring element (of diameter 31 pixels). The disc diameter is set larger than the base diameter of each target feature.

Relevant parameter settings of each method were optimized for fair comparison throughout the experiment.

Table 1 ▶ lists the measured CIR values for each contrast enhancement image (with *R*_C_ and *R*_N_ set to 11 and 41, respectively). Because it generates a remarkably higher contrast than either conventional method, the proposed method achieves significantly higher CIR values.

In the proposed method, each target structure is clearly extracted from the background. Additionally, as is evident from the histogram of the image enhanced by the proposed method [Fig. 2(*d*) ▶, right panel], the intensities of the target areas are homogeneously distributed. The histogram peaks corresponding to target structures are distinct from each other as well as from the background.

The local contrast value [equation (15)] is the difference in average greyscale intensity between the target structures and the surrounding region. The more homogeneous the intensity distribution in the target structures, the higher the local contrast. Consequently, the CIR increases.

The most distinctive property of the proposed method is that it enhances the target features while ensuring homogeneous intensity distribution. This property improves the visibility of subtle mass lesions and reduces unwanted background information even in real biomedical images. It also enables automatic segmentation of target structures. In segmentation processing, an image is divided into multiple segments (*e.g.* multiple target structures and backgrounds) by classifying pixels according to histogram thresholds. Thus, successful segmentation relies upon the presence of distinct peaks in the histograms corresponding to target structures. Consequently, the proposed method may be applicable to segmentation pre-processing.

### 3.2. Application results

**Figure 3:**
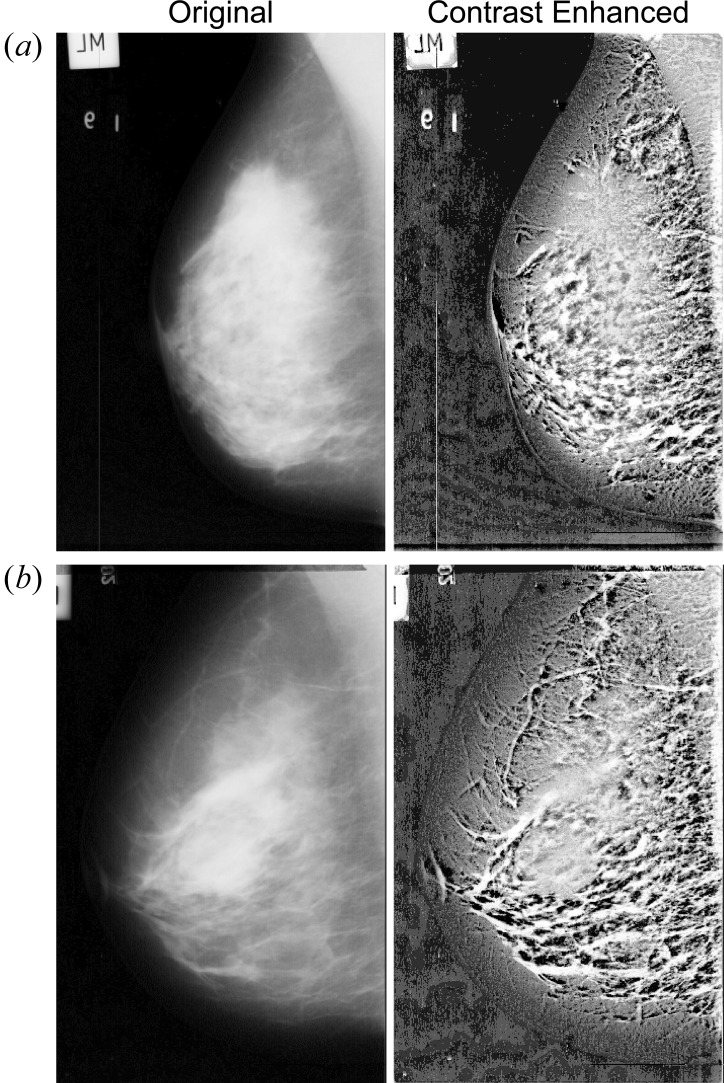
Enhancement of mammographic images by the proposed method. Left-hand panels: original mammographic images [(*a*) mdb003, (*b*) mdb315]. Right-hand panels: contrast enhanced images obtained by the proposed method.

Fig. 3 ▶ illustrates mammographic images enhanced by the proposed method. The original images [Fig. 3 ▶, left-hand panel, (*a*) mdb003, (*b*) mdb315] are shown at half their original size. Thus, spatial resolution is 400 µm pixel^−1^. In the right-hand panel of this figure, the mammary gland structure has been enhanced using a disc-shaped structuring element of diameter 31 pixels (or 12.4 mm). The target structure (mammary gland) is rendered clearly evident, and improved contrast is achieved throughout the entire breast region. Furthermore, the breast border contour (the skin–air interface, which tends to be low-contrast and scarcely visible) is easily identified.

**Figure 4:**
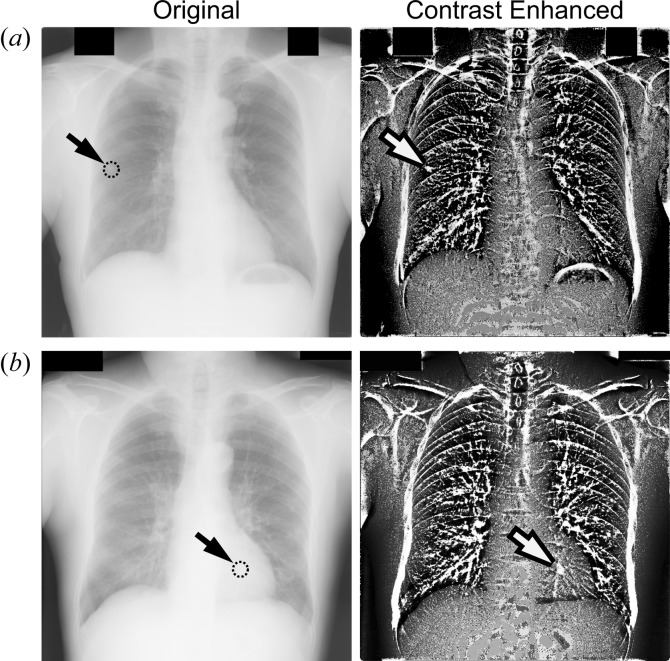
Enhancement of chest radiographic images by the proposed method. Left-hand panels: original chest radiographic images [(*a*) JPCLN80, (*b*) JPCLN152]. The arrow in each image indicates a nodule. Right-hand panels: contrast enhanced images obtained by the proposed method.

Fig. 4 ▶ shows chest radiographic images enhanced by the proposed method. The original images [Fig. 4 ▶, left-hand panel, (*a*) JPCLN080, (*b*) JPCLN152] have been resized to 512 × 512 pixels (spatial resolution is 0.7 mm pixel^−1^). The arrow indicates the nodule position in each image. As before, a disc-shaped structuring element (of diameter 31 pixels; in this case corresponding to 21.7 mm) was used. In the enhanced images (Fig. 4 ▶, right-hand panel), the nodules are more clearly evident while the surrounding tissues are suppressed.

To avoid amplification of any noise in the original image, a noise reduction process is necessary before applying the proposed contrast enhancement method. In general, morphological smoothing can be used as an effective noise reduction technique. RMP opening [equation (7)], RMP closing [equation (8)] and RMP-based morphological smoothing filter (Kimori *et al.*, 2007 ▶; Kimori, 2011 ▶) are also used for this purpose. Since these filters are constructed by RMP, edge-preserving smoothing can be accomplished. The effectiveness of the noise reduction processing has been evaluated in our previous studies (Kimori *et al.*, 2007 ▶, 2010 ▶).

## 4. Conclusion

Image contrast enhancement is one of the most important stages in image processing. It reveals and highlights various structural features that are scarcely detectable to the human eye. This paper presents a new RMP-based method that enhances contrast in biomedical images. The method involves three steps: (i) selective extraction of target features by top-hat operations, (ii) greyscale modification of the top-hat images, and (iii) combination of greyscale modified top-hat images and the original image. This method specifically enhances target structures from complex surroundings and uneven background intensity.

The superior performance of the proposed method was demonstrated by comparing its CIR value with those of two conventional methods. The effectiveness and usefulness of the method were demonstrated by application to medical images.

## Figures and Tables

**Table 1:** Contrast improvement ratio (CIR) for different contrast enhancement methods (MSR: multiscale retinex; CLAHE: contrast-limited adaptive histogram equalization; Proposed: proposed method)

	MSR	CLAHE	Proposed
CIR	4.663	0.095	12.111

## References

[bb1] Chisada, S., Okamoto, H., Taniguchi, Y., Kimori, Y., Toyoda, A., Sakaki, Y., Takeda, S. & Yoshiura, Y. (2011). *Dev. Biol.* **359**, 82–94. 10.1016/j.ydbio.2011.08.027 21925159

[bb2] Galatsanos, N. P., Segall, C. A. & Katsaggelos, A. K. (2003). *Encyclopedia of Optical Engineering*, edited by R. G. Driggers, pp. 388–402. New York: Taylor and Francis.

[bb3] Gonzalez, R. C. & Woods, R. E. (2008). *Digital Image Processing*, 3rd ed. New Jersey: Prentice Hall.

[bb4] Jobson, D. J., Rahman, Z. & Woodell, G. A. (1997). *IEEE Trans. Image Process.* **6**, 965–976. 10.1109/83.597272 18282987

[bb5] Kimori, Y. (2011). *J. Clin. Bioinforma.* **1**, 33. 10.1186/2043-9113-1-33 PMC3275547 22177340

[bb6] Kimori, Y., Baba, N. & Morone, N. (2010). *BMC Bioinformatics*, **11**, 373. 10.1186/1471-2105-11-373 PMC2914730 20615231

[bb7] Kimori, Y., Katayama, E., Morone, N. & Kodama, T. (2011). *J. Struct. Biol.* **176**, 1–8. 10.1016/j.jsb.2011.07.007 21801838

[bb8] Kimori, Y., Oguchi, Y., Ichise, N., Baba, N. & Katayama, E. (2007). *Ultramicroscopy*, **107**, 25–39. 10.1016/j.ultramic.2006.04.012 16777331

[bb9] Kobatake, H. & Yoshinaga, Y. (1996). *IEEE Trans. Med. Imaging*, **15**, 235–245. 10.1109/42.500062 18215905

[bb10] Meyer, F. (1979). *J. Histochem. Cytochem.* **27**, 128–135. 10.1177/27.1.438499 438499

[bb11] Oh, J. & Hwang, H. (2010). *Int. J. Control Autom. Syst.* **8**, 857–861.

[bb12] Pisano, E. D., Cole, E. B., Hemminger, B. M., Yaffe, M. J., Aylward, S. R., Maidment, A. D., Johnston, R. E., Williams, M. B., Niklason, L. T., Conant, E. F., Fajardo, L. L., Kopans, D. B., Brown, M. E. & Pizer, S. M. (2000). *Radiographics*, **20**, 1479–1491. 10.1148/radiographics.20.5.g00se311479 10992035

[bb22] Pizer, S. M., Amburn, E. P., Austin, J. D., Cromartie, R., Geselowitz, A., Greer, T., Romeny, B. T. H., Zimmerman, J. B. & Zuiderveld, K. (1987). *Comput. Vis. Graph. Image Process.* **39**, 355–368.

[bb13] Reljin, B., Milosević, Z., Stojić, T. & Reljin, I. (2009). *Folia Histochem. Cytobiol.* **47**, 525–532. 10.2478/v10042-009-0076-1 20164042

[bb14] Serra, J. (1982). *Image Analysis and Mathematical Morphology.* London: Academic Press.

[bb15] Shiraishi, J., Katsuragawa, S., Ikezoe, J., Matsumoto, T., Kobayashi, T., Komatsu, K., Matsui, M., Fujita, H., Kodera, Y. & Doi, K. (2000). *Am. J. Roentgenol.* **174**, 71–74. 10.2214/ajr.174.1.1740071 10628457

[bb16] Soille, P. (1999). *Morphological Image Analysis.* Berlin: Springer-Verlag.

[bb17] Suckling, J., Parker, J., Dance, D. Astley, S., Hutt, I., Boggis, C., Ricketts, I., Stamatakis, E., Cerneaz, N., Kok, S., Taylor, P., Betal, D. & Savage, J. (1994). *Exerpta Medica Intl Congr. Ser.* **1069**, 375–378.

[bb18] Thackray, B. D. & Nelson, A. C. (1993). *IEEE Trans. Med. Imaging*, **12**, 385 -392. 10.1109/42.241865 18218430

[bb19] Wang, D. & Vagnucci, A. H. (1981). *Comput. Vis. Graph. Image Process.* **24**, 363–381.

[bb20] Wang, Y. P., Wu, Q., Castleman, K. R. & Xiong, Z. (2003). *IEEE Trans. Med. Imaging*, **22**, 685–693. 10.1109/TMI.2003.812255 12846437

[bb21] Yasuda, T., Oda, S., Li, Z., Kimori, Y., Kamei, Y., Ishikawa, T., Todo, T. & Mitani, H. (2012). *Cell Death Dis.* **3**, e395. 10.1038/cddis.2012.133 PMC3481122 23034330

